# Black–White Color Metaphors of Justice: Two Experiments on Justice as a Legal Value

**DOI:** 10.3390/bs16030367

**Published:** 2026-03-05

**Authors:** Shuhui Xu, Weiwei Sun, Kaihang Zhang

**Affiliations:** 1 Department of Psychology, Wenzhou University, Wenzhou 325035, China; 2 Institute of Higher Education, Wenzhou University, Wenzhou 325035, China; 3 College of Education, Wenzhou University, Wenzhou 325035, China; 4 Wenzhou Research Center for Family Tradition and Family Education, Wenzhou 325000, China

**Keywords:** justice as legal value, legal socialization, color metaphor, conceptual metaphor, Stroop paradigm, translation-matching

## Abstract

Color metaphors may shape how people mentally represent abstract legal values such as justice and thereby influence legal socialization and law-related cognition. We tested whether black/white color terms are metaphorically linked to justice conceived specifically as a legal value, and whether these linkages vary with task demands. In two preregistered experiments that controlled for affective valence, word frequency, and semantic relatedness, Experiment 1 employed a Stroop-style lexical-judgment task with law-relevant terms and found faster responses to justice-related (legal) words than to injustice-related words and higher accuracy for white-colored stimuli, but no reliable color × meaning interaction—suggesting the absence of an automatic color–justice congruency effect during early, automatic processing. Experiment 2 used a translation-matching paradigm in which participants selected black or white translations for unfamiliar foreign words; here, participants systematically matched justice-related (legal) items with white and injustice-related items with black at rates above chance, revealing explicit color–justice associations. Together, the results point to a robust mental linkage of white with justice as a legal value, while black–injustice mappings emerge primarily under explicit selection demands. These findings suggest that black/white color metaphors organize law-related moral cognition but are flexibly activated depending on cognitive task and processing level.

## 1. Introduction

Justice occupies a central position, not only in political philosophy and jurisprudence, but also in social psychology and cognitive science. As a normative and evaluative concept, justice encompasses both institutional dimensions (procedural and distributive) and moral dimensions (integrity and impartiality) at the semantic level (Sandel, 2009). Within the Chinese linguistic and cultural context, justice is often defined as “fairness and integrity; absence of partiality,” reflecting both concerns regarding the reasonableness of resource allocation and references to the moral character of actors and the legitimacy of social institutions (Fan & Guo, 2011; W. Li, 2015).

Beyond philosophical and cultural analyses, a growing body of developmental research suggests that sensitivity to fairness and justice emerges early in life. Studies employing looking-time and resource-distribution paradigms indicate that infants as young as 12–15 months form expectations of equal distribution and evaluate agents who violate distributive fairness more negatively (Schmidt & Sommerville, 2011; Sloane et al., 2012). By toddlerhood, children not only detect unequal distributions but also selectively reward-fair actors and prefer prosocial distributors (Kenward & Dahl, 2011). These findings suggest that core components of justice evaluation are supported by early-developing socio-cognitive mechanisms.

Recent research has begun to integrate fairness evaluation with intergroup perception. Toddlers’ and preschoolers’ fairness judgments are embedded within broader social-cognitive processes, including expectations about reciprocity, sensitivity to coalition signals, and attention to group-based cues. For example, toddlers selectively intervene in response to fair versus unfair individuals, suggesting that early fairness sensitivity is not merely perceptual but motivational (Ziv et al., 2021). Moreover, infants by 15 months of age form expectations about positive and negative reciprocity between strangers, indicating that early moral expectations are responsive to social contingencies that may shape group-directed evaluations (Jin et al., 2024).

Of particular relevance to the present research are studies linking distributive-fairness evaluations to emerging intergroup markers, including visible skin-color cues. Geraci et al. (2024) found that toddlers attributed fair distributions more frequently to Black rather than White distributors, suggesting that sensitivity to intergroup coalition cues may already be present in early childhood. Broader reviews further indicate that racial and ethnic awareness, as well as implicit bias, can be detected early and are often acquired through observational social learning in everyday environments (Meltzoff & Gilliam, 2024; Schmidt & Rakoczy, 2023). Collectively, this body of evidence supports three conclusions: (a) core fairness computations emerge in infancy; (b) infants and toddlers can incorporate social-categorical cues when forming inferences about distributive actors; and (c) early exposure and social learning processes shape how fairness and race/skin-color cues become associated across development. Accordingly, analyses of adult justice perceptions that neglect these developmental origins risk overlooking formative processes that contribute to intergroup justice judgments in adulthood.

Taken together, integrating developmental findings with socio-legal perspectives enables a more comprehensive theoretical framework in which justice is conceptualized both as an early-emerging evaluative capacity and as a construct shaped by cultural and institutional contexts. Such an integrative approach clarifies the developmental roots of justice perception while situating adult evaluations within a broader lifespan trajectory.

## 2. Theoretical Framework

### 2.1. Historical and Philosophical Perspectives on Justice

The deep-rooted ideas of justice in traditional Chinese culture provide an important historical context for contemporary understandings. Confucian thought espouses principles such as “tiānxià wéi gōng” (“the world belongs to the public”) (*Lǐjì*, Lǐyùn chapter) and the admonition “bù huàn guǎ ér huàn bù jūn” (literally “do not worry about scarcity but about unequal distribution”) (*Lúnyǔ*, Jìshì chapter), both of which emphasize distributive fairness and political legitimacy. Legalist writings—exemplified by the claim of strict, undifferentiated punishment in the *Shāng Jūn Shū* (*Book of Lord Shang*)—pursue institutionalized, procedural forms of justice (Fang, 2015). These intellectual strands not only underpin traditional governance philosophies but also inform contemporary articulations of justice within Chinese socialist core values.

From the standpoint of political philosophy, Rawls (1971) famously defines justice as “the first virtue of social institutions” and advances a theory of justice as fairness. Subsequent interpretations in Chinese scholarship further elaborate Rawls’ framework, particularly its emphasis on procedural impartiality and the protection of basic liberties (Zhou, 2014). By contrast, Hayek (1976) critiqued the coherence of “social justice,” while acknowledging that just rules contribute to the maintenance of spontaneous order; his account emphasizes rule-based and negative conceptions that guard against severe forms of injustice. Multidisciplinary scholarship has since expanded empirical and conceptual analyses of justice. For example, Colquitt (2001) differentiated four justice dimensions—distributive, procedural, interpersonal, and informational—whereas Du et al. (2010) used factor analysis to show that lay conceptions of justice in China may reflect additional culturally specific dimensions (e.g., rights, equity, remedy, retribution, equality, and equal sharing). Together, these works underscore that justice is a multilevel construct profoundly shaped by cultural context.

### 2.2. From Abstract Concept to Sensory Metaphor

Justice is a highly abstract evaluative construct within the broader domain of legal-value cognition, which encompasses individuals’ representations of fairness, legitimacy, and institutional norms (Xu & Yan, 2022). Unlike concrete legal knowledge—such as statutes or procedures—justice primarily indexes culturally mediated normative values rather than directly observable referents.

Conceptual metaphor theory posits that abstract concepts are often understood via mappings from perceptual or embodied source domains (Lakoff & Johnson, 1980). Complementary perspectives from embodied cognition and perceptual symbol theory argue that low-level perceptual schemas (e.g., light–dark, up–down) can be recruited to structure higher-order representations of morality, authority, and social evaluation (Barsalou, 1999). Empirical work indicates that color and luminance cues can bias moral evaluations across tasks and cultures, making the light–dark axis a plausible perceptual scaffold for moral values such as justice (Meier et al., 2004; Yin & Ye, 2014).

Still, the notion that justice is reducible to a single perceptual metaphor is contested. Legal scholars have noted variations in how metaphorically embedded different legal concepts are: some legal categories (e.g., “law” or “intellectual property”) are richly metaphorical, whereas justice as an abstract evaluative value resists reduction to a single perceptual source (Winter, 2001; Larsson, 2013; Wojtczak & Witczak-Plisiecka, 2024). Experimental grounding studies suggest that legal concepts can partly rely on sensorimotor and social experiences while remaining shaped by cultural and institutional practices (Roversi et al., 2017; Borghi et al., 2022). These findings imply that perceptual-moral mappings must be interpreted within developmental, linguistic, and institutional contexts rather than treated as universal laws.

Taken together, these perspectives motivate a nuanced hypothesis for the present study: color cues along a black–white dimension may serve as one perceptual affordance that influences moral-semantic processing, but such mappings are probabilistic, context-dependent, and mediated by cultural learning. The current research does not attempt to reduce justice to a sensory proxy; rather, it tests whether perceptual cues can bias conceptual access and evaluative judgments in measurable ways. This approach is consistent with contemporary accounts of early moral cognition that emphasize interactions among domain-general perceptual biases, culturally transmitted symbolic meanings, and nascent fairness expectations (Surian et al., 2025).

### 2.3. Empirical Evidence for Color–Moral Metaphors

A substantial empirical literature shows that color cues influence moral and value judgments. Across cultures, white or high-luminance stimuli are more readily associated with positive moral attributes (e.g., “good,” “trustworthy,” “fair”), whereas black or low-luminance stimuli are associated with negative or impure connotations (Meier et al., 2004; Sherman & Clore, 2009). Experimental manipulations—such as background contrast, Stroop-type tasks, and situational judgment paradigms—further demonstrate that visual contrast and the luminance of bias-evaluative responses (Yin & Ye, 2014; Zarkadi & Schnall, 2013). In Chinese contexts, white backgrounds have been reported to promote honesty and prosocial behavior, consistent with a white–good/black–bad mapping (Song et al., 2018).

Recent work highlights neural, cultural, and developmental moderators of these effects. Neuroimaging and neuropsychological studies implicate distributed networks, including prefrontal and occipital regions, in mediating the influence of color on moral judgment (Gan et al., 2022). Implicit-association studies across cultures show robust white-positive and black-negative associations that are, nevertheless, shaped by local cultural practices (Kawai et al., 2023). Corpus analyses in Chinese reveal that metaphorical uses of bái (“white”) vary with pragmatic and cultural context, underscoring the role of language and social experience (Dou et al., 2023).

Developmental evidence further elucidates the origins and propagation of these mappings. Toddlers selectively attribute fair distributions to individuals based on visible group markers, including skin color, pointing to early intergroup fairness biases (Geraci et al., 2024). Core moral concepts such as fairness and impartiality are detectable in infancy and may provide building blocks for adult moral cognition (Surian et al., 2025). Collectively, these findings suggest that adult color–moral associations arise from an interplay among early social sensitivities, domain-general valence biases, and culturally transmitted symbolic systems.

Importantly, the strength and direction of color–moral associations are context-sensitive. Cross-cultural comparisons and developmental studies both indicate that perceptual cues interact with social categorization and group biases; consequently, color–moral metaphors reflect situated cognitive and social processes rather than fixed or deterministic mappings (Kawai et al., 2023; Dou et al., 2023).

## 3. The Present Research: Justice-Specific Color Metaphors and Legal Socialization

Prior research offers initial evidence for black–white mappings with justice and fairness concepts. Embodied and metaphoric studies generally find that white is associated with justice while black is associated with injustice, although results vary across paradigms and task types (Chen, 2014; Q. Li, 2018). These findings indicate that color–justice mappings exist, but their directionality, symmetry, and psychological robustness remain unresolved.

At the same time, some scholars have questioned whether justice is appropriately characterized as a perceptual metaphor. Legal theorists argue that justice is fundamentally a socio-institutional construct shaped by norms, law, and governance rather than a concept readily reducible to perceptual mappings (Bauer, 1976; Hirschtick, 1976; Miller, 2021). Philosophical analyses likewise warn that metaphorical accounts risk oversimplifying justice’s multi-layered cognitive and institutional foundations (Ricœur, 1977). Integrating these critiques with empirical work, an empirical investigation of black–white–justice mappings can clarify the degree to which perceptual metaphors reflect intrinsic cognitive structure versus culturally mediated symbolic associations.

Framing the question within a legal socialization perspective highlights its applied significance. Individuals internalize representations of justice that help shape trust in legal institutions, compliance motivations, and civic behavior; these representations may be supported or biased by perceptual and embodied cues (Tyler, 2003; Tyler & Trinkner, 2017). Thus, understanding how black–white color mappings relate to justice judgments can illuminate cognitive mechanisms relevant to the development of law-abiding attitudes.

To address these issues, we ran two complementary behavioral experiments grounded in conceptual-metaphor and embodied-cognition frameworks. Experiment 1 used a Stroop-style color-word interference task to probe automatic mapping effects between black/white colors and justice/injustice concepts by measuring response times and accuracy. Experiment 2 employed a translation-matching paradigm in which participants chose unfamiliar foreign words presented in black or white; by minimizing explicit semantic retrieval, this task probed implicit influences of color on conceptual matching. Stimuli in both experiments were controlled for affective valence and word frequency to reduce potential confounders.

This study focuses on three core questions: (1) Do stable black–white mappings exist for justice versus injustice concepts? (2) If mappings exist, do they manifest consistently across automatic processing (Experiment 1) and less explicit decision contexts (Experiment 2)? (3) What implications do such mappings have for legal socialization, particularly for the formation of trust in judicial institutions and law-abiding attitudes?

## 4. Study 1

Grounded in conceptual metaphor theory—which conceives metaphors as systematic cross-domain mappings whereby individuals use concrete, embodied source domains to organize and understand abstract target domains—this experiment investigated whether black/white color cues form stable metaphorical linkages with the concept of justice, and whether such color cues affect the efficiency of processing justice-related versus injustice-related lexical items. Justice, as a prototypical abstract concept, may rely on perceptual dimensions (e.g., black–white) in its cognitive representation.

We implemented a modified Stroop paradigm in which participants performed a semantic classification task, judging each presented word as “just” (justice-related) or “unjust” (injustice-related). The experiment manipulated lexical meaning (justice vs. injustice) and stimulus color (white vs. black), and established congruent conditions (white–justice, black–injustice) and incongruent conditions (white–injustice, black–justice).

**Hypothesis** **1.** *If white↔justice and black↔injustice constitute metaphorically consistent mappings, congruent trials should show a processing advantage (shorter reaction times and higher accuracy), whereas incongruent trials should induce cognitive conflict, producing slower responses and lower accuracy.*

### 4.1. Method

#### 4.1.1. Participants

This study received approval from the Institutional Review Board and was conducted in accordance with the ethical principles of the Declaration of Helsinki. Written informed consent was obtained from all participants prior to the study.

Sixty undergraduate students were initially recruited (15 males, 45 females; age range 18–22 years). All participants were right-handed, had normal or corrected-to-normal vision, and reported no color blindness, color weakness, or history of reading disorders. To ensure data quality, participants with overall accuracy below 90% were excluded, and individual trials with incorrect responses or reaction times exceeding ±3 standard deviations from each participant’s mean were discarded. The final analytic sample comprised 51 participants.

#### 4.1.2. Materials

Materials consisted of 16 justice-related words and 16 injustice-related words, selected from the *Modern Chinese Dictionary* and relevant prior literature. A separate pretest was conducted with 34 independent participants (not involved in the main experiment) to validate the semantic valence and familiarity of the items. Pretest raters evaluated each word on justice perception (1 = extremely unjust, 5 = extremely just) and familiarity (1 = very unfamiliar, 5 = very familiar).

Pretest results indicated that justice words were rated significantly above the scale midpoint (*M* = 3.97, *SD* = 0.90; *t*(33) = 6.73, *p* < 0.001), whereas injustice words were rated significantly below the midpoint (*M* = 2.11, *SD* = 1.29; *t*(33) = −4.02, *p* < 0.001). The two word sets differed significantly on perceived justice (*t*(33) = 8.45, *p* < 0.001). Familiarity ratings did not differ significantly between the sets (justice words: *M* = 4.02, *SD* = 0.98; injustice words: *M* = 4.06, *SD* = 1.00). These results indicate clear semantic separation between the categories and satisfactory matching on familiarity.

#### 4.1.3. Design

The experiment used a 2 (lexical meaning: justice vs. injustice) × 2 (word color: white vs. black) within-subjects design. Dependent variables were reaction time for correct semantic-classification trials (measured in milliseconds) and classification accuracy (percentage correct).

#### 4.1.4. Procedure

The experiment took place in a controlled computer laboratory. Experimental scripts were programmed and run using E-Prime 2.0. Stimuli were presented in SimSun font (Chinese “No. 2” size) centered on the screen in either black (grayscale 100%) or white (grayscale 0%), against a mid-gray background (grayscale 50%) to balance visual contrast.

**Figure 1:**
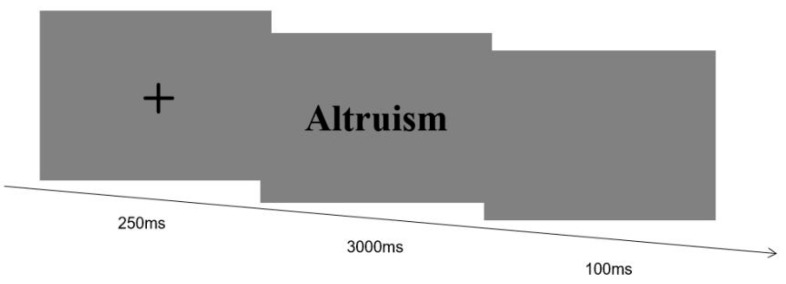
Trial timeline for Experiment 1.

Each trial proceeded as follows: a central fixation cross (“+”) appeared for 250 ms, followed immediately by the target word. Participants were instructed to classify the word’s meaning within 3000 ms as accurately and quickly as possible—pressing the “F” key for “justice” and the “J” key for “injustice.” Failure to respond within the 3000 ms window was recorded as an error. The formal experiment comprised 64 trials, with an equal and randomized distribution of trials across the experimental conditions. Ten practice trials preceded the formal session to ensure participants understood the task. See Figure 1.

### 4.2. Results

The descriptive statistics for participants’ reaction times and accuracy rates across the experimental conditions are shown in Table 1 and Table 2. With respect to reaction time, justice-related words displayed in white yielded the shortest mean reaction time (*M* = 811.06 ms, *SD* = 333.37), while injustice-related words displayed in black resulted in the longest mean reaction time (*M* = 927.98 ms, *SD* = 416.98). Regarding accuracy, justice-related words presented in white produced the highest mean accuracy rate (*M* = 0.98, *SD* = 0.151), whereas injustice-related words presented in black showed the lowest mean accuracy rate (*M* = 0.94, *SD* = 0.240).

Separate 2 (word color: black vs. white) × 2 (lexical meaning: justice vs. injustice) repeated-measures ANOVAs were conducted on reaction time and accuracy.

For reaction time, there was a significant main effect of lexical meaning, F(1, 50) = 74.276, *p* < 0.001, partial η^2^ = 0.598, indicating that participants responded significantly faster to justice-related words than to injustice-related words. The main effect of word color was not significant, F(1, 50) = 0.769, *p* = 0.381, partial η^2^ = 0.015, suggesting no reliable RT difference between words displayed in white versus black. The interaction between lexical meaning and word color was also non-significant, F(1, 50) = 1.867, *p* = 0.172, partial η^2^ = 0.036.

For accuracy, there was a significant main effect of word color, F(1, 50) = 20.859, *p* < 0.001, partial η^2^ = 0.294, indicating that words presented in white were classified more accurately than words presented in black. The main effect of lexical meaning on accuracy was not significant, F(1, 50) = 0.031, *p* = 0.861, partial η^2^ = 0.001. The lexical meaning × word color interaction for accuracy was likewise non-significant, F(1, 50) = 0.771, *p* = 0.380, partial η^2^ = 0.015.

### 4.3. Discussion

Study 1 used a Stroop-style semantic classification task to test whether black and white color cues map onto justice and injustice concepts. Two primary results emerged. First, there was a robust semantic main effect: justice-related words were classified faster than injustice-related words, suggesting greater accessibility or facilitation for justice semantics during retrieval or decision stages (Rozin & Royzman, 2001; Vaish et al., 2008). Second, a reliable color main effect appeared in accuracy: stimuli presented in white were classified more accurately than those presented in black, consistent with the idea that color can act as a salient visual cue that sharpens semantic judgments (Elliot & Maier, 2014; Kawai et al., 2023). The predicted congruency interaction, wherein white paired with justice and black paired with injustice would yield superior performance, failed to reach statistical significance, although the white paired with justice condition showed a descriptive advantage.

Several factors may account for the null interaction. First, metaphorical mappings between perceptual cues and abstract moral concepts are likely subtle, and Stroop-style tasks are sensitive to processing speed and stimulus control, which may limit their ability to detect small-cross-domain effects (MacLeod, 1991). Second, negatively valenced items often elicit deeper, more elaborative processing, which can attenuate the influence of peripheral visual cues and reduce the likelihood that color-based congruency effects appear in reaction-time measures (Rozin & Royzman, 2001). Third, low-level perceptual parameters such as luminance, contrast, and lexical characteristics can dilute weak cross-domain mappings in interference paradigms (MacLeod, 1991). That we found a color effect on accuracy but not a reliable congruency interaction suggests that perceptual salience may provide a general facilitative influence without necessarily producing strong semantic congruency mapping. Related work showing context-dependent color effects on social evaluation further implies that color cues exert contingent external influences on judgments rather than universal semantic mappings (Hill & Barton, 2005).

Viewed developmentally, these results are compatible with accounts that place core moral concepts such as fairness and impartiality among early-emerging and central elements of the moral cognitive system (Surian et al., 2025). The observed facilitation for justice-related terms dovetails with the view that fairness-related constructs occupy privileged cognitive status, perhaps reflecting continuity from nascent fairness expectations in infancy to elaborated adult representations. At the same time, the absence of a robust Stroop congruency effect indicates that perceptual cues such as color do not automatically instantiate moral mappings at the level of lexical competition. Instead, perceptual-valence mappings, for example, light associated with positive valence, may provide a broad affective scaffold that becomes linked to explicit moral concepts through cultural, linguistic, and institutional learning.

Overall, the pattern of semantic facilitation for justice, a white-related accuracy advantage, and the lack of stable bidirectional congruency supports a layered model of moral representation. In this model, early affective–associative biases and domain-general valence systems provide initial scaffolding, while culturally elaborated symbolic meanings and language-mediated reasoning shape explicit moral concepts. Corpus and cross-cultural analyses corroborate this perspective by showing that color semantics are culturally modulated and pragmatically variable (Dou et al., 2023; Kawai et al., 2023). Thus, rather than revealing a fixed perceptual-moral equivalence, our findings point to probabilistic, context-sensitive mappings that interact with developmental and cultural processes to influence adult moral semantic processing.

## 5. Study 2

Study 2, like Study 1, is grounded in conceptual metaphor theory and aims to examine whether perceptual black–white cues form stable metaphorical linkages with the abstract concept of justice. Diverging from the Stroop paradigm used in Experiment 1, Experiment 2 adopts a translation-matching paradigm (inspired by Yin & Ye, 2014). By asking participants to choose between two foreign (non-native) translation options presented in black or white, this indirect task probes implicit color–concept associations while minimizing explicit semantic retrieval of the foreign items.

**Hypothesis** **2.** *If black/white colors map metaphorically onto justice/injustice, participants should preferentially choose the white-presented translation when the Chinese target is a justice-related word and preferentially choose the black-presented translation when the Chinese target is an injustice-related word. The behavioral index is therefore the difference in the proportion of white-choice responses between the two lexical conditions.*

### 5.1. Method

#### 5.1.1. Participants

This study was approved by the Institutional Review Board and conducted in accordance with the ethical principles outlined in the Declaration of Helsinki. Written informed consent was obtained from all participants prior to data collection.

Sixty undergraduate students (15 males, 45 females; aged 18–22 years) were recruited for the study. All participants were right-handed, had normal or corrected-to-normal vision, and reported no color vision deficiencies or reading disorders. In addition, all participants confirmed that they had no prior knowledge of or exposure to the Russian language.

Participants received a brief explanation of the general purpose of the task before completing the experiment. Individuals who failed to follow task instructions or exhibited extreme or invalid response patterns were excluded from the analyses. The final sample comprised 55 valid participants.

#### 5.1.2. Materials

Materials were identical to those in Study 1: 16 justice-related and 16 injustice-related Chinese words. Each Chinese target was paired with two Russian translation candidates, only one of which was the correct translation. All Russian items were validated via back-translation and pretested on an independent sample to confirm that Chinese undergraduates could not reliably infer their meanings; this step ensured that foreign-word semantics would not overtly influence choices. The two translation alternatives differed only in color—one rendered in black (grayscale 100%) and the other in white (grayscale 0%)—while font, font size, screen position, and contrast were held constant so that color served as the only diagnostic cue. The detailed materials are provided in the Supplementary Materials.

#### 5.1.3. Design

A single-factor within-subjects design was used, with lexical type (justice vs. injustice) as the independent variable. The dependent variable was the proportion of trials in which a participant selected the white translation. To control for side and response biases, the left/right positions of black and white translations were fully counterbalanced across trials, and the mapping of response keys to left/right choices (left key selects left item; right key selects right item) was counterbalanced across participants.

#### 5.1.4. Procedure

**Figure 2:**
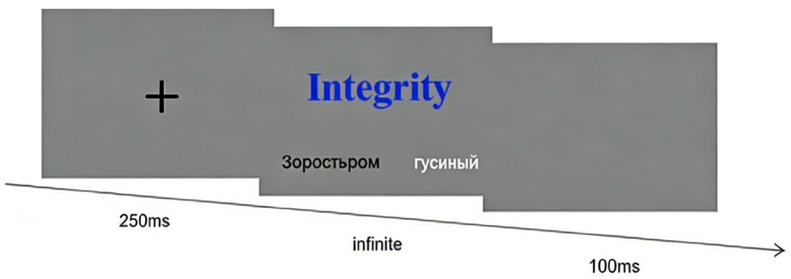
Trial timeline for Experiment 2. Зоростьром (n.)—Zoroastrianism; an ancient monotheistic religion founded by Zoroaster in Persia. гусиный (adj.)—“goose”; denotes something related to geese (e.g., goose feather, goosebumps).

Testing took place in a controlled computer laboratory. The experiment was programmed and ran in E-Prime 2.0. Each trial proceeded as follows: a central fixation cross (“+”) appeared for 250 ms; then, a blue Chinese target word (presented in “size 1” font) appeared at the top of the screen, and two Russian translation options (presented in “size 2” font) appeared side-by-side beneath it against a mid-gray background (grayscale 50%). Participants indicated their choice by pressing the “F” key for the left option or the “J” key for the right option. There was no time limit for responses. The formal test comprised 32 trials (16 justice, 16 injustice) presented in randomized order; black/white item positions were balanced across trials. Six to eight practice trials preceded the formal session to ensure that participants understood the task. See Figure 2.

#### 5.1.5. Data Recording and Analysis

The experiment recorded participants’ choices (left vs. right) and reaction times for each trial. The primary dependent measures were, for each participant, the proportion of white selections in the justice condition and the proportion of white selections in the injustice condition. If a “white–justice/black–injustice” metaphorical mapping is present, the probability of selecting a white-associated term should be significantly higher in the justice condition than in the injustice condition.

To test this hypothesis, a repeated-measures analysis of variance (ANOVA) was conducted with word meaning (justice vs. injustice) as the within-subjects independent variable and the proportion of white-translated word selections as the dependent variable. Effect sizes (partial η^2^) and 95% confidence intervals were reported to evaluate the magnitude and precision of the observed effects.

To complement the participant-level analysis and fully utilize the trial-level data, item-level analyses were also conducted. Specifically, one-sample *t*-tests (against a chance level of 50%) were performed separately for justice-related and injustice-related words. For these analyses, effect sizes (Cohen’s d) and 95% confidence intervals were reported.

The analytic pipeline documented the number of excluded or invalid trials and the reasons for exclusion, reported the proportion of missing data (if any), and presented descriptive statistics (condition means and standard deviations or standard errors) alongside the inferential results.

### 5.2. Results

Choice frequencies for the 32 trials were converted to percentages to index color–matching preferences; descriptive statistics are shown in Table 3. When the target word was a justice-related item, participants chose the white translation on 58.12% of trials; when the target was an injustice-related item, the white-translation choice rate was 42.71%. Correspondingly, the black-translation choice rates were 41.88% for justice items and 57.29% for injustice items.

A repeated-measures analysis of variance (ANOVA) was conducted with word meaning (justice vs. injustice) as the within-subjects independent variable and the proportion of white-translated word selections per participant as the dependent variable. The analysis revealed a significant main effect of word meaning, F(1, 54) = 16.636, *p* < 0.001, partial η^2^ = 0.236, indicating that participants’ color selections differed significantly between the two word categories. The effect size was in the medium-to-large range.

To further examine this pattern, an item-level analysis was performed. One-sample *t*-tests (against a chance level of 50%) were conducted separately on the selection rates of white-translated words for each word type (each group *n* = 16). For justice-related words, the selection rate of white-translated options was significantly above chance, t(15) = 4.163, *p* < 0.01, with a large effect size (Cohen’s d = 1.04). In contrast, for injustice-related words, the selection rate was significantly below chance, t(15) = −2.418, *p* < 0.05, corresponding to a medium effect size (Cohen’s d = 0.60).

Taken together, the findings of Study 2 provide robust evidence of color–concept congruency in the translation-matching task. Participants systematically associated justice-related Chinese words with white translations and injustice-related Chinese words with black translations.

### 5.3. Discussion

Study 2 used a translation-matching paradigm to test whether black–white color cues map onto justice and injustice concepts. Participants were significantly more likely to choose the translation displayed in white when the target word was justice-related, and to choose the translation displayed in black when the target word was injustice-related. This pattern held at both the participant and item levels, providing direct behavioral evidence for the hypothesized white → justice and black → injustice mappings.

Because participants generally could not identify the literal meanings of the foreign translation items, their selections were driven primarily by intuition rather than by explicit semantic retrieval. This feature makes the paradigm well suited for capturing the automatic influence of nonverbal cues on concept matching: choices here likely reflect unconscious or semi-conscious associations rather than deliberative reasoning. Classic cross-cultural work shows that light/white and dark/black are widely associated with positive and negative values, respectively, offering a broad explanation for color-driven intuitive choices (Adams & Osgood, 1973). Color may also activate embodied purity/contamination representations that modulate moral judgment—for example, the influence of physical cleansing on moral perception (Zhong & Liljenquist, 2006). At the same time, corpus and cross-cultural analyses indicate that the magnitude and even direction of color–value associations are moderated by cultural context and pragmatic usage (Kawai et al., 2023; Dou et al., 2023).

Several caveats complicate interpretation. First, part of the effect could reflect low-level visual salience or contrast: white translations may have been more visually salient against the mid-gray background. Future studies should equate luminance and contrast across conditions (or explicitly model these parameters) to rule out simple perceptual confounders. Second, the mapping appears asymmetric: our data provide more consistent support for a white → positive/justice cue than for a robust black → negative/injustice cue. This asymmetry aligns with prior work showing that positive light associations are often stronger and more stable than analogous dark associations, which tend to be more context sensitive. Third, although the translation-matching task reveals choice preferences, it does not resolve temporal dynamics; we cannot determine from these data whether associations emerge during early automatic activation or during later decision stages. Combining tasks sensitive to latency (e.g., the Implicit Association Test) with time-resolved physiological measures (e.g., event-related potentials) would help dissociate early automatic activation from deliberative processing (Greenwald et al., 1998; Luck, 2014).

Interpreted developmentally, the present results dovetail with evidence that rudimentary fairness expectations and sensitivity to social cues emerge early in life (Surian et al., 2025). Empirical work shows that young children recruit visible social markers, such as skin color, when reasoning about distribution and reciprocity (Geraci et al., 2024; Jin et al., 2024). Such developmental data suggest that adult color–moral mappings may arise through a long-term integration of early perceptual–affective biases with socially and culturally mediated learning and symbolism.

Overall, Study 2 provides convergent behavioral evidence that color can function as an embodied cue influencing abstract concept matching. However, the mappings we observe are best characterized as probabilistic and context dependent rather than fixed or deterministic.

## 6. Implications

Justice, as a core legal value, occupies a central place in legal socialization. Justice cognition encompasses not only evaluations of rules and procedures but also constitutes an affective foundation for perceptions of legitimacy and social trust. The goal of legal socialization is therefore not merely the transmission of rule knowledge, but the internalization of legal norms as stable behavioral guides through institutional interaction and symbolic experience (Tyler & Blader, 2003; Lind & Tyler, 1988). Procedural experiences—feelings of respect, transparency, and opportunities for participation—consistently shape institutional legitimacy and voluntary compliance (Sunshine & Tyler, 2003; Mazerolle et al., 2013).

The present research examined whether abstract legal values such as justice can be anchored by perceptual symbolic cues, specifically black–white color contrast. Results showed that black and white colors function as visual metaphors that influence justice-related cognition. Specifically, the white → justice mapping was robust across both automatic processing and explicit choice preferences, whereas the black → injustice mapping proved more contingent on task context and deeper cognitive engagement. This pattern supports embodied cognition and conceptual metaphor accounts, demonstrating that bodily experience, including visual perception, can organize abstract concepts (Barsalou, 1999; Kövecses, 2010), while also indicating that such mappings may operate differently at distinct cognitive stages (Gawronski & Bodenhausen, 2006).

From a mechanistic perspective, color-to-concept mappings plausibly operate via two complementary pathways. The first is an associative–automatic pathway in which recurring co-occurrences of color and evaluative language in culture produce stimulus–affect associations (Meier et al., 2004; Sherman & Clore, 2009). The second is a propositional–control pathway whereby semantic integration and explicit judgment recruit cognitive resources to endorse, modify, or override these associative tendencies (Gawronski & Bodenhausen, 2006; Bargh & Chartrand, 1999). Hu et al. (2025) find that abstract legal cognition significantly and negatively predicts university students’ unlawful risk-taking, with this protective effect strongest among individuals high in need for cognitive structure. These findings highlight that interventions targeting the development of abstract legal cognition—such as reinforcing justice-related conceptual mappings—can cultivate self-regulated, law-abiding behavior, providing a direct rationale for integrating perceptual and symbolic cues into legal socialization programs. Together, these mechanisms account for the stability of the white → justice mapping and the greater contextual sensitivity of the black → injustice mapping.

Framed within legal socialization, these mechanisms point to two practical implications. First, visual and symbolic interventions—such as systematically incorporating light, transparent design elements in public legal communications or courtroom materials—may reinforce intuitive perceptions of justice and thereby strengthen perceptions of procedural fairness and institutional trust (Tyler & Blader, 2003). Second, symbolic design must accompany substantive institutional improvements; symbolic cues can amplify the impact of genuine procedural enhancements (e.g., transparency, participation, respectful treatment) but cannot substitute for them (Sunshine & Tyler, 2003; Mazerolle et al., 2013).

In sum, this study offers an embodied-cognition account of legal socialization by showing that visual metaphors can serve as auxiliary tools in legal education and rule-of-law communication. The effectiveness of such interventions, however, depends on their coordination with substantive institutional practices and real procedural experiences.

## 7. Limitations and Future Directions

Several limitations of the present study warrant consideration, and future research should extend this line of inquiry in several important directions.

First, the generalizability of the findings is constrained by the characteristics of the sample. Participants were drawn from a relatively homogeneous population within a specific cultural and geographic context. Accordingly, caution is warranted when extending these results to other age groups, cultural backgrounds, socioeconomic strata, or institutional settings. Future studies should test the proposed mechanisms in more diverse samples, including children, adolescents, older adults, and individuals from different cultural regions. Cross-cultural comparative designs would be especially informative in determining whether color–justice associations reflect culturally specific symbolic systems or more universal cognitive tendencies (Adams & Osgood, 1973).

Second, the symbolic meanings of black and white are culturally embedded and context dependent. In some sociocultural settings, these colors may convey divergent or even contradictory connotations. Systematic cross-cultural research would help disentangle culturally acquired associations from potentially domain-general perceptual-moral mappings.

Third, the present study did not systematically assess or control for participants’ affective states. Because transient mood can influence both perceptual processing and moral evaluation (Forgas, 2017), future research should incorporate validated mood assessments or experimental mood inductions to rule out potential affective confounders.

Fourth, the reliance on behavioral indicators limits conclusions regarding the temporal dynamics and implicit cognitive mechanisms underlying the observed effects. Future investigations would benefit from integrating methods with greater temporal resolution, such as event-related potentials, as well as implicit measures such as the Implicit Association Test (Greenwald et al., 1998), to distinguish automatic from controlled processes and to clarify the cognitive architecture of color–justice metaphors (Gawronski & Bodenhausen, 2006).

Finally, extending this research beyond laboratory paradigms to applied judicial or educational contexts would enhance ecological validity. Field or intervention studies conducted in real institutional settings would enable researchers to evaluate the practical effectiveness, boundary conditions, and longer-term consequences of color-based visual metaphors for institutional trust, perceptions of procedural justice, and law-abiding behavior.

## 8. Conclusions

Synthesizing the findings of the two experiments, the present study demonstrates that there exists a metaphorical psychological association between the concepts of justice and the colors black and white, and that this association is asymmetrical. The link between white and justice proved relatively stable, emerging both at the level of automatic processing and in explicit judgment. By contrast, the association between black and injustice was more contingent, depending heavily on task context and the depth of cognitive processing. These results align with the basic “white–good, black–bad” metaphorical schema proposed in conceptual metaphor theory (Johnson & Lakoff, 2002) and further suggest that color metaphors extend beyond linguistic expressions to exert influence on social cognition and value judgments.

This study provides empirical support for the metaphorical representation of justice, thereby expanding the intersection between color psychology and legal social cognition. It also underscores that the construction and transmission of legal values, to some extent, relies on unconscious, embodied cognitive frameworks rooted in everyday experiences.

## Figures and Tables

**Table 1:** Reaction times (ms) and standard deviations in word judgments.

Word Color	Word Meaning	
	Justice-Related Words	Injustice-Related Words
White	811.06 ± 333.37	901.71 ± 342.00
Black	817.42 ± 318.88	927.98 ± 416.98

**Table 2:** Accuracy rates and standard deviations in word judgments.

Word Color	Word Meaning	
	Justice-Related Words	Injustice-Related Words
White	0.98 ± 0.151	0.95 ± 0.226
Black	0.97 ± 0.166	0.94 ± 0.240

**Table 3:** Percentage of word selections by participants.

Word Color	Word Meaning	
	Justice-Related Words	Injustice-Related Words
White	58.12%	42.71%
Black	41.88%	57.29%

## Data Availability

The raw data supporting the conclusions of this article will be made available by the authors on request.

## Supplementary Materials

The following supporting information can be downloaded at: https://www.mdpi.com/article/10.3390/bs16030367/s1, Table S1: Justice-related words (Chinese); Table S2: Injustice-related words (Chinese); Table S3: Russian words used in Experiment 2.
