# Supplementary material for: Black–White Color Metaphors of Justice: Two Experiments on Justice as a Legal Value
Source: Behav Sci (Basel). 2026 Mar 5;16(3):367. doi: 10.3390/bs16030367 (PMC13023880; doi:10.3390/bs16030367)
Supplement: Supplementary file 1 [file behavsci-16-00367-s001.zip › behavsci-4114422-supplementary.pdf]

**Supplementary Materials: Stimulus Words Used in the Experiments**

**Table S1.** Justice-Related Words (Chinese).

| No. | Word | English Translation |
|---|---|---|
| 1 | 公正 | justice (noun) |
| 2 | 公平 | fair/equitable |
| 3 | 平均 | average |
| 4 | 均摊 | distribute equally |
| 5 | 无私 | selfless |
| 6 | 正派 | decent |
| 7 | 刚直 | upright |
| 8 | 清廉 | incorruptible |
| 9 | 秉公 | uphold justice |
| 10 | 公允 | fair |
| 11 | 公信 | public trust |
| 12 | 公道 | fairness |
| 13 | 平等 | equality |
| 14 | 正直 | integrity |
| 15 | 均分 | share equally |
| 16 | 均衡 | balance |

**Table S2.** Injustice-Related Words (Chinese).

| No. | Word | English Translation |
|---|---|---|
| 1 | 偏私 | biased |
| 2 | 护短 | be overprotective of / cover up one's fault |
| 3 | 袒护 | be partial to / shield |
| 4 | 隐瞒 | conceal |
| 5 | 不等 | inequality |
| 6 | 不均 | uneven |
| 7 | 偏心 | partiality / bias |
| 8 | 偏见 | prejudice |
| 9 | 歧视 | discrimination |
| 10 | 私心 | self-interest |
| 11 | 偏爱 | partiality |
| 12 | 偏袒 | bias |
| 13 | 徇私 | favoritism |
| 14 | 不公 | unjust |
| 15 | 包庇 | harbor / cover up for |
| 16 | 偏激 | provoke conflict |

**Table S3.** Russian Words Used in Experiment 2.

| No. | Russian Word | English Translation |
|---|---|---|
| 1 | справедливость | justice (noun) |
| 2 | справедливый | just (adj.) |
| 3 | среднее | average |
| 4 | распределить поровну | distribute equally |
| 5 | бескорыстный | selfless |
| 6 | порядочный | decent |
| 7 | твёрдый | firm |
| 8 | честный | honest |
| 9 | соблюдать справедливость | uphold justice |
| 10 | справедливый | fair |
| 11 | официальное письмо | public trust |
| 12 | справедливость | fairness |
| 13 | равенство | equality |
| 14 | прямота | directness / frankness |
| 15 | разделить поровну | share equally |
| 16 | уравновешивать | balance |
| 17 | пристрастный | biased |
| 18 | укоренившийся | entrenched |
| 19 | покровительствовать | patronize |
| 20 | скрывать | conceal |
| 21 | неравенство | inequality |
| 22 | неравномерный | uneven |

| No. | Russian Word | English Translation |
|---|---|---|
| 23 | эксцентриситет | eccentricity |
| 24 | предвзятость | prejudice |
| 25 | дискриминация | discrimination |
| 26 | личные интересы | self-interest |
| 27 | пристрастие | partiality |
| 28 | пристрастность | bias |
| 29 | фаворитизм | favoritism |
| 30 | несправедливый | unjust |
| 31 | покровительствовать | show favoritism |
| 32 | возбуждать | provoke |

Note: Russian words 1–16 correspond to justice-related meanings, and words 17–31 correspond to injustice-related meanings. English translations are provided for reference.
